# Integrating hepatology with addiction care for inpatients with alcohol use disorder reduces future liver-related events

**DOI:** 10.1097/HC9.0000000000000780

**Published:** 2025-07-29

**Authors:** Paul George, Chantelle Marshall, Wei Zhang, Russell Goodman, Michael Butler, Suraj J. Patel, Mack Mitchell, Esperance Schaefer, Jay Luther

**Affiliations:** 1 Department of Internal Medicine, Massachusetts General Hospital, Harvard Medical School, Boston, Massachusetts, USA; 2 MGH Alcohol Liver Center, Massachusetts General Hospital, Harvard Medical School, Boston, Massachusetts, USA; 3 Division of Gastroenterology, Massachusetts General Hospital, Harvard Medical School, Boston, Massachusetts, USA; 4 Division of Gastroenterology, University of Texas at Southwestern, Dallas, Texas, USA; 5 Division of Gastroenterology, Brigham and Women’s Hospital, Harvard Medical School, Boston, Massachusetts, USA

**Keywords:** alcohol use, liver disease, multidisciplinary

## Abstract

**Background:**

Strategies to identify patients with early alcohol-associated liver disease (ALD), prior to the development of liver-related decompensated events, and promote alcohol therapy engagement in these patients are urgently needed to stem the rising tide of mortality associated with ALD.

**Methods:**

We compared the rate of incident liver-related decompensating events in hospitalized patients with alcohol use disorder (AUD) seen either by an integrated hepatology and addiction care approach or addiction care alone at 2 academic medical centers. Cox proportional hazards regression model and a Kaplan–Meier analysis were used.

**Findings:**

An integrated approach of hepatology and addiction care is associated with a reduced likelihood of future liver-related decompensating events in hospitalized patients with AUD. This finding correlated with an increased uptake of medical alcohol therapy and a reduced likelihood of an alcohol-associated readmission. Integrated hepatology and addiction care for hospitalized AUD patients may help reduce the progression of ALD.

## INTRODUCTION

Alcohol-associated liver disease (ALD) is the most common cause of death from excessive alcohol use, and this mortality will likely double over the next 2 decades.^1^ Many with ALD are diagnosed after suffering life-threatening liver-related complications, when therapies are less effective.^2^ Early identification is critical to initiate therapies that can slow or even halt disease progression and can provide liver transplant committees with information to guide transplant decisions in patients with end-stage ALD.

To address this deficiency, we created a first-of-its-kind inpatient consultation service, the Alcohol LIVer Evaluation (ALIVE) team, for hospitalized patients with alcohol use disorder (AUD). This hepatology-led team works with the inpatient addiction medicine team and evaluates patients with AUD and no prior history of cirrhosis or alcohol-associated hepatitis. The ALIVE team: (1) educates patients on the harmful effects of alcohol on the liver, (2) evaluates for subclinical ALD with elastography followed by liver biopsy when clinically indicated, (3) promotes the benefits and hepatic safety of formal AUD therapy in preventing progression of ALD, and (4) encourages follow-up in a hepatology clinic.^3^ We have previously shown that the addition of ALIVE to addiction consultation improves hepatic fibrosis detection, alcohol therapy engagement, and hepatology follow-up compared to patients seen by the addiction consult team alone.^4^^,^^5^ However, the impact of ALIVE on preventing progression of liver disease to an advanced form, defined by the presence of a decompensating liver-related event, remains unknown. In this work, we examined the association of the ALIVE evaluation with the future development of liver-related complications in hospitalized patients with AUD. We hypothesized that early identification of ALD patients (those with hepatic fibrosis but no prior episodes of decompensation) and engagement in alcohol therapy with ALIVE would be associated with reduced liver disease progression.

## METHODS

The cohort group was collected prospectively at Massachusetts General Hospital (MGH) and included patients hospitalized between June 2020 and May 2024, with a diagnosis of AUD, who were evaluated by the ALIVE service and the addiction medicine consult service. The control group was collected retrospectively at Brigham and Women’s Hospital (BWH), another tertiary center within our health care system (Mass General Brigham), and included patients hospitalized between January 2019 and January 2022, with a diagnosis of AUD, and an evaluation by the inpatient addiction service. Exclusion criteria included: (1) a previous diagnosis of cirrhosis or evaluation by a hepatologist; (2) a MELD score of ≥11, as values in this range define the presence of at least moderate acute alcohol-associated hepatitis and would require formal hepatology consultation; and (3) a bilirubin ≥3.0 mg/dL. The primary outcome was the development of a liver-related decompensating event (ascites, bleeding esophageal varices, HE, HCC, or hepatorenal syndrome). We used a Cox proportional hazards regression model to account for the follow-up duration, generating adjusted hazard ratios (aHRs) for the development of liver-related complications, accounting for variables in Supplemental Table S1, http://links.lww.com/HC9/C60. We used a Kaplan–Meier analysis to examine the proportion of patients over time who developed a liver-related decompensating event after the index hospitalization.

## RESULTS

In total, 423 patients were included in the ALIVE plus addiction medicine group and 96 patients in the control arm (Supplemental Figure S1, http://links.lww.com/HC9/C61). Demographic, clinical, and biochemical measures, including the presence of metabolic risk factors and comorbid viral hepatitis, were similar between each group (Supplemental Table S1, http://links.lww.com/HC9/C60). Further, values for noninvasive tests for advanced hepatic fibrosis (FIB-4, FibroTest, and APRI scores) were similar between groups. Patients were followed for an average of 2.22 years in the ALIVE plus addiction medicine group and 3.94 years in the addiction care alone group.

**FIGURE 1:**
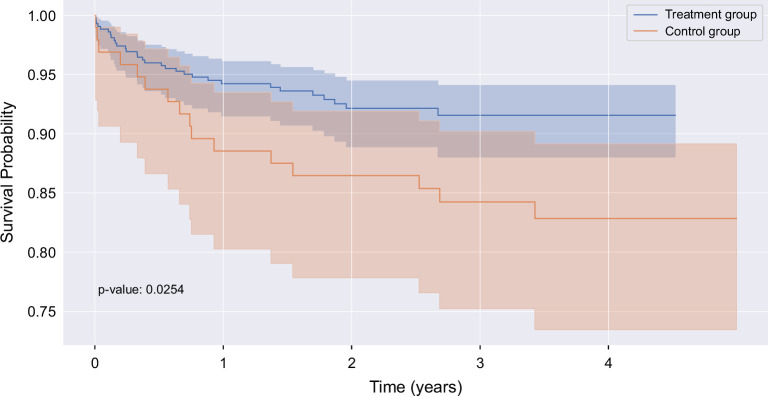
ALIVE plus addiction care is associated with reduced future liver-related decompensating events. The Kaplan–Meier curve shows the likelihood of staying free of the development of liver-related decompensating events (*Y*-axis), defined by the presence of ascites, variceal bleeding, HE, hepatorenal syndrome, and HCC, over time (*X*-axis) in patients evaluated by ALIVE and addiction medicine (treatment group) compared to controls. Abbreviation: ALIVE, Alcohol LIVer Evaluation.

We found that 20% of patients evaluated by ALIVE were found to have newly diagnosed advanced hepatic fibrosis (F2 or greater) or subclinical cirrhosis by elastography and/or liver biopsy at the index hospitalization. These tests were not performed in the control group at index hospitalization, as is the current standard of care for hospitalized patients with AUD and no clear evidence of active liver disease. Patients seen by ALIVE were more likely to be discharged on alcohol pharmacotherapy and had lower alcohol-associated hospital readmission rates at 30 and 90 days (18% vs. 32%, *p*=0.005, and 35% vs. 49%, *p*=02, respectively) compared to patients seen by addiction medicine alone. Regarding liver-related outcomes, we found that patients evaluated by ALIVE plus addiction medicine were less likely to develop a liver decompensating event during the follow-up period (aHR: 0.50, 95% CI: 0.27–0.90, *p*=0.03) (Figure 1). ALIVE patients were less likely to develop variceal bleeding (aHR: 0.13, 95% CI: 0.03–0.51, *p*<0.005) and HE (aHR: 0.24, 95% CI: 0.08–0.73, *p*=0.01). There was no difference in the incidence of ascites development between the groups.

## DISCUSSION

Integrating addiction and hepatology services in a multidisciplinary clinic has been shown to improve liver-related outcomes in ambulatory patients with ALD.^6^ Additionally, while previous care modes added addiction treatment to the hepatology care of patients with severe liver disease,^7^ our study is the first to suggest that earlier integration of hepatology with addiction medicine for hospitalized patients may improve AUD pharmacotherapy uptake, improve readmission rates, and reduce progression of ALD to advanced stages. Given the current lack of effective therapies for patients with advanced ALD, identifying and engaging patients with earlier stages of disease offers a durable treatment strategy to combat the predicted rising tide of ALD-related mortality. The inclusion of addiction psychiatry to this model will likely further improve outcomes by providing behavioral or psychotherapeutic remedies to address the comorbid psychological illnesses often seen in ALD patients.

Limitations in our work include using different methods to identify populations, which may create a poorer comparison, variability in patient resources despite similarities in these hospitals, and a lack of data on psychiatric comorbidities or AUD severity that could introduce bias. While the smaller number of patients in the control group may be a function of BWH having roughly seventy percent of the number of inpatient beds as MGH, it also may be related to certain unmeasured inherent differences between each hospital. Finally, alcohol abstinence rates, which represent the likely mechanism for our observed association, were difficult to define in both groups, given the high frequency of missing data.

## Supplementary Material
