# Supplementary material for: Integrating hepatology with addiction care for inpatients with alcohol use disorder reduces future liver-related events
Source: Hepatol Commun. 2025 Jul 29;9(8):e0780. doi: 10.1097/HC9.0000000000000780 (PMC12306700; doi:10.1097/HC9.0000000000000780)
Supplement: Supplementary file 1 [file hc9-9-e0780-s001.docx]

**Supplementary Table 1. Patient Demographics**

| **Characteristic** | **Control (N = 96)** | **ALIVE (N = 423)** | **P-value** |
| --- | --- | --- | --- |
| Age, Median (IQR), y | 53.5 (40.75 - 59.25 = 18.5) | 48.0 (39.0 - 58.5 = 19.5) | 0.11144 |
| Sex |  |  |  |
| Male, n (%) | 66 (68) | 326 (77.07) | 0.10131 |
| Female, n (%) | 30 (31) | 97 (22.93) |  |
| Race |  |  |  |
| White, n (%) | 60 (62) | 293 (69.27) | .00121 |
| Black, n (%) | 22 (22) | 41 (9.69) |  |
| Other/Unknown, n (%) | 14 (14) | 89 (21.04) |  |
| Ethnicity |  |  |  |
| Hispanic | 12 (13) | 86 (20.33) | 0.14083 |
| Non-Hispanic | 82 (85) | 322 (76.12) |  |
| Primary Language |  |  |  |
| English, n (%) | 84 (87) | 347 (82.03) | 0.2551 |
| Non-English, n (%) | 12 (12) | 76 (17.97) |  |
| BMI, Median (IQR), kg/m^2^ | 25.0 (22.31 - 28.25 = 5.94) | 26.82 (23.94 - 30.86 = 6.92) | 0.00157 |
| ALT, Median (IQR), u/L | 50.0 (24.0 - 96.0 = 72.0) | 55.0 (32.0 - 101.0 = 69.0) | 0.0993 |
| AST, Median (IQR), u/L | 103.0 (46.0 - 204.0 = 158.0) | 100.0 (47.0 - 184.0 = 137.0) | 0.82457 |
| ALP, Median (IQR), u/L | 115.0 (83.0 - 175.0 = 92.0) | 106.0 (80.0 - 140.0 = 60.0) | 0.10923 |
| TB, Median (IQR), mg/dL | 0.7 (0.5 - 1.5 = 1.0) | 0.8 (0.5 - 1.3 = 0.8) | 0.82433 |
| Albumin, Median (IQR), mg/dL | 4.2 (3.9 - 4.6 = 0.7) | 4.2 (3.8 - 4.6 = 0.8) | 0.48522 |
| Hgb, Median, g/dL (IQR) | 13.3 (11.7 - 14.4 = 2.7) | 13.5 (11.95 - 14.9 = 2.95) | 0.19084 |
| MCV, Median, fL (IQR) | 92.5 (88.5 - 99.0 = 10.5) | 93.5 (88.1 - 97.5 = 9.4) | 0.81673 |
| PLT, Median (IQR), K/uL | 177.0 (115.0 - 270.0 = 155.0) | 183.0 (122.25 - 249.0 = 126.75) | 0.63636 |
| INR, Median (IQR) | 1.1 (1.0 - 1.1 = 0.1) | 1.1 (1.0 - 1.2 = 0.2) | 0.87046 |
| PT, Median (IQR), s | 13.6 (13.2 - 14.3 = 1.1) | 13.7 (12.9 - 14.6 = 1.7) | 0.88428 |
| HbA1c, Median (IQR) | 5.3 (4.95 - 5.8 = 0.85) | 5.4 (5.0 - 5.9 = 0.9) | 0.30995 |
| FIB4, Median (IQR) | 3.69 (1.94 - 8.31 = 6.37) | 3.38 (1.75 - 6.55 = 4.8) | 0.14228 |
| Fibrotest, Median (IQR) | 0.5 (0.21 - 0.75 = 0.54) | 0.32 (0.15 - 0.56 = 0.41) | 0.19214 |
| APRI, Median (IQR) | 0.87 (0.42 - 3.18 = 2.76) | 1.4 (0.52 - 3.45 = 2.93) | 0.21536 |
| MELD, Median (IQR) | 9.0 (7.0 - 13.0 = 6.0) | 7.91 (6.57 - 10.43 = 3.86) | 0.13125 |
| HAV IgG |  |  |  |
| Positive, n(%) | 18 (18) | 162 (38.3) | 0.38984 |
| Negative, n(%) | 23 (24) | 147 (34.8) |  |
| HBcAb |  |  |  |
| Positive, n(%) | 6 (6) | 24 (5.67) | 0.39196 |
| Negative, n(%) | 48 (50) | 330 (78.01) |  |
| HBsAb |  |  |  |
| Positive, n(%) | 16 (16) | 140 (33.1) | 0.54977 |
| Negative, n(%) | 47 (49) | 230 (54.37) |  |
| HBsAg |  |  |  |
| Positive, n(%) | 0 (0) | 7 (1.65) | 0.0785 |
| Negative, n(%) | 67 (70) | 367 (86.76) |  |
| HCV ab |  |  |  |
| Positive, n(%) | 17 (18) | 63 (14.89) | 0.2778 |
| Negative, n(%) | 60 (63) | 326 (77.06) |  |

Abbreviations: ALT, alanine transaminase; APRI, ast to platelet ratio index; AST, aspartate transaminase; BMI, body mass index; FIB4, fibrosis-4 index; HAV, hepatitis a virus; HbA1c, hemoglobin A1c; HBcAb, hepatitis b core antibody; HBsAb, hepatitis b surface antibody; HBsAg, hepatitis b surface antigen; HCV ab, hepatitis c virus antibody; Hgb, hemoglobin; IgG, immunoglobulin g; INR, international normalized ratio; IQR, interquartile range; MCV, mean corpuscular volume; MELD, model for end-stage liver disease; PLT, platelet; PT, prothrombin time; TB, total bilirubin.
