# Supplementary figures and images for: Integrating hepatology with addiction care for inpatients with alcohol use disorder reduces future liver-related events
Source: Hepatol Commun. 2025 Jul 29;9(8):e0780. doi: 10.1097/HC9.0000000000000780 (PMC12306700; doi:10.1097/HC9.0000000000000780)

**Supplementary Figure 1. Flow diagram of patient selection**

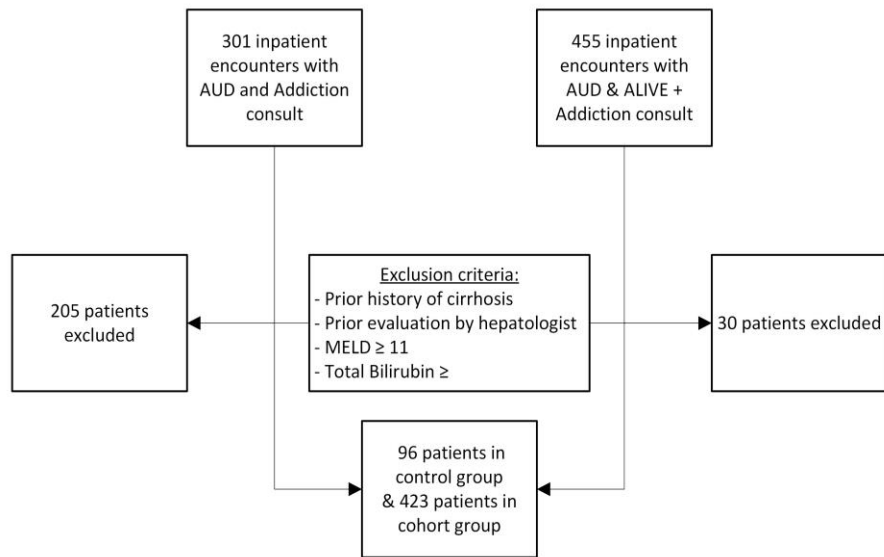

Supplement: Supplementary file 2 [file hc9-9-e0780-s002.pdf]
